# Tranexamic acid for spontaneous intracerebral hemorrhage: hematoma control without clinical benefits? A meta-analysis of RCTs

**DOI:** 10.1007/s00210-025-04473-5

**Published:** 2025-07-28

**Authors:** Mahmoud M. Elhady, Eslam Mohammed Rabea, Samah Bahy Mohammed Ebaed, Manar Adel, Moustafa Z. Elattar, Mahmoud Eleisawy, Ahmed A. Lashin, Ahmed A. Elfeky, Mohamed Hesham Gamal, Mohamed Sayed Zaazouee

**Affiliations:** 1 Faculty of Medicine, Benha University, Qalubiya, Egypt; 2 Faculty of Medicine, Alexandria University, Alexandria, Egypt; 3 Department of Clinical Pharmacology, Faculty of Medicine, Benha University, Benha, Egypt; 4 Faculty of Clinical Pharmacy, Tanta University, Gharbia, Egypt; 5 Anatomy & Embryology Department, Faculty of Medicine, Benha University, Qalubiya, Egypt; 6 Ophthalmology Department, Benha University Hospitals, Qalubiya, Egypt; 7 Faculty of Medicine, Beni Suef University, Beni Suef, Egypt; 8 Faculty of Pharmacy, Tanta University, Gharbia, Egypt; 9 Faculty of Medicine, Al-Azhar University, Assiut, Egypt

**Keywords:** Spontaneous intracranial hemorrhage, Tranexamic acid, Hematoma expansion, Meta-analysis

## Abstract

Spontaneous intracerebral hemorrhage (sICH) is a serious form of stroke associated with high mortality and poor functional outcomes. Hematoma expansion critically affects prognosis, necessitating early intervention. Tranexamic acid (TXA), an antifibrinolytic, has an uncertain role in sICH. This meta-analysis synthesizes evidence from randomized controlled trials (RCTs) to evaluate the efficacy and safety of TXA in sICH. Comprehensive searches of PubMed, Scopus, Web of Science, and Cochrane Library identified RCTs comparing intravenous TXA to placebo in sICH, focusing on hematoma expansion. Data on functional outcomes, mortality, thromboembolic events, and quality of life were extracted. Cochrane RoB2 assessed the quality of studies. RevMan 5.4 was used for data analysis, employing fixed-effects models. Subgroup analyses were conducted based on time windows and patient subgroups. Eight RCTs (2974 patients) were included. TXA significantly reduced 24-h hematoma volume (MD =  − 1.17 mL, 95% CI =  − 1.97 to − 0.36), particularly when administered within 8 h (MD =  − 1.71 mL, 95% CI =  − 2.68 to − 0.74). However, odds of expansion, functional outcomes, 90-day mortality, thromboembolic events, and quality of life were similar between groups. Another subgroup analysis based on study populations revealed a significant effect of TXA on hematoma volume in studies involving sICH without a specific subgroup classification. However, no significant effect was observed in other subgroups, including sICH with spot sign, hypertensive ICH, and NOAC-associated ICH. TXA has been shown to reduce hematoma expansion in sICH, particularly when administered within 8 h of symptom onset, without a corresponding increase in thromboembolic events. However, this benefit has not translated into significant improvements in functional outcomes, mortality, or quality of life. As such, current evidence does not support the routine use of TXA in sICH, and further high-quality research is warranted.

**Supplementary Information:**

The online version contains supplementary material available at 10.1007/s00210-025-04473-5.

## Introduction

Spontaneous intracerebral hemorrhage (sICH) describes the non-traumatic form of ICH in which blood extravasation occurs spontaneously within the brain parenchyma (Aguilar and Freeman 2010). The annual incidence reaches around 25 per 100,000, with high early mortality (40% at 1 month) and poor long-term outcomes in survivors (Fallenius et al. 2019). The case fatality rate is extremely high, with around 60% of patients dying within 1 year of the event (Faghih-Jouybari et al. 2021). Among survivors, only 20% achieve independence within 6 months. ICH is strongly linked to risk factors like chronic hypertension, advanced age, cerebral amyloid angiopathy, leukoaraiosis, prior ICH, renal failure, and antithrombotic (antiplatelet and anticoagulant) therapy (Sallinen et al. 2020). Key predictors of unfavorable outcomes in ICH include low Glasgow Coma Scale (GCS) score, abnormal blood pressure (BP) levels, large hematoma size, and the presence of midline shift (Al-Alawi et al. 2023).

Decreasing the risk of hematoma expansion is the primary target for mitigating the consequences of ICH (Kazui et al. 1996). The guidelines emphasize early and intensive BP control as a key strategy in managing acute ICH, balancing the risks of hematoma expansion and cerebral hypoperfusion (Greenberg et al. 2022; Anderson et al. 2013). Other suggested strategies to limit hematoma expansion include hemostatic therapies like platelet transfusion, recombinant factor VII, fresh frozen plasma (FFP), and antifibrinolytic drugs. However, the evidence does not support their routine use, as they have shown no significant benefit and may even pose risks, such as the harmful effects reported with platelet transfusion. However, the use of antifibrinolytics in sICH has been an ongoing debate (Al-Shahi Salman et al. 2018). Tranexamic acid (TXA), an antifibrinolytic agent, has been shown to reduce bleeding progression in traumatic ICH, but this has not been reflected in statistically significant clinical improvement (Zehtabchi et al. 2014). Investigating the role of intravenous TXA in sICH has shown controversial conclusions. The largest trial (TICH-2), including 2325 patients, reported that TXA did not significantly improve functional status at 3 months compared to placebo in patients with sICH. Although tranexamic acid reduced early mortality and serious adverse events, it did not lead to a significant decrease in deaths within 90 days (Sprigg et al. 2018). Previous meta-analyses stated that early TXA administration could significantly reduce the incidence of hematoma expansion in sICH, mainly if treated within 4.5 h and those at high risk for hematoma expansion. However, no significant differences were demonstrated in functional outcomes or mortality rates between the TXA and placebo groups (Yassi et al. 2024; Jiao et al. 2021; Guo et al. 2021). Newer RCTs have been published with controversial results (Yassi et al. 2024; Hollingworth et al. 2024; Arumugam et al. 2023). This meta-analysis seeks to consolidate the available evidence from RCTs to provide an updated assessment of the role of TXA in sICH, focusing on identifying specific subgroups to optimize its use.

## Methods

This study followed the PRISMA guidelines for systematic reviews and meta-analyses (Page et al. 2021).

### Search strategy

We searched PubMed, Scopus, the Cochrane Library, and Web of Science (WOS) using the following search terms: (Intracerebral OR Subarachnoid OR Intraparenchymal OR Parenchymal OR Intraventricular OR intra-axial OR Intracranial OR brain OR Cerebral OR Stroke) AND (hemorrhage OR bleeding OR hematoma) AND (Tranexamic acid OR Cyklokapron OR AMCHA OR AMCA OR t-AMCHA OR trans-4-(Aminomethyl)cyclohexanecarboxylic Acid OR Transamin OR Ugurol OR KABI 2161 OR Spotof OR Amchafibrin OR Anvitoff OR Lysteda OR Exacyl OR Evana OR antifibrinolytic) AND (random*). Details of search strategies and search results across the four databases are demonstrated in Supplementary Table 1. Retrieved records were exported to Endnote for duplicate removal and subsequently screened by two independent reviewers based on titles and abstracts. Studies deemed relevant underwent a thorough full-text review to determine eligibility according to our prespecified criteria.

### Eligibility criteria

We employed the PICOS framework to identify eligible studies: Population: patients with sICH; Intervention: tranexamic acid (TXA); Control: placebo; Outcomes: safety and efficacy outcomes, with hematoma expansion as the primary outcome of interest; Study design: only RCTs were eligible. We excluded studies that were non-English, non-RCTs, or focused on traumatic intracerebral hemorrhage or subarachnoid hemorrhage.

### Data extraction and risk of bias

Two independent reviewers extracted relevant data from the included studies. Information on study characteristics, including protocol number, location, sample size, intervention details, study populations, time window of TXA administration, follow-up durations, and primary outcomes, was extracted and tabulated. We compiled baseline data, including demographics (age, gender), comorbidities, blood pressure, Glasgow Coma Scale (GCS) score, National Institutes of Health Stroke Scale (NIHSS) scores, Modified Rankin Score (mRS), and CT angiography (CTA) spot sign positivity. All efficacy and safety outcomes were organized in an Excel spreadsheet. Our primary outcome of interest was hematoma expansion, assessed by two measures: absolute increase in hematoma volume (mL) and the odds of expansion, defined as a 33% relative increase or 6 mL absolute volume increase. Secondary outcomes included assessments of functional outcomes (mRS and NIHSS scores), 90-day mortality rates, major thromboembolic event odds, and health-related quality of life metrics (EQ-5D Health Utility Score, EQ Visual Analog Scale, and Zung Depression Scale). The quality of the included studies was evaluated using the Cochrane RoB2 tool (Sterne et al. 2019).

### Statistical analysis

The analysis was conducted using RevMan version 5.4. A fixed-effects model was adopted as all analyses showed no statistical heterogeneity. Heterogeneity was quantified using the *I*^2^ statistic, with values over 50% and a *p* value below 0.1, indicating significant heterogeneity (“9.5.2 Identifying and measuring heterogeneity 2025). Using a 95% confidence interval (CI), risk ratios (RR) and mean differences (MD) were calculated for dichotomous and continuous outcomes, respectively. Subgroup analyses were conducted based on the time windows of TXA administration (within 4.5 h, 8 h, and 24 h). Further subgrouping was performed based on study populations, including sICH, sICH with a spot sign, hypertensive ICH, and novel oral anticoagulant (NOAC)-associated ICH.

## Results

### Search results

**Fig. 1:**
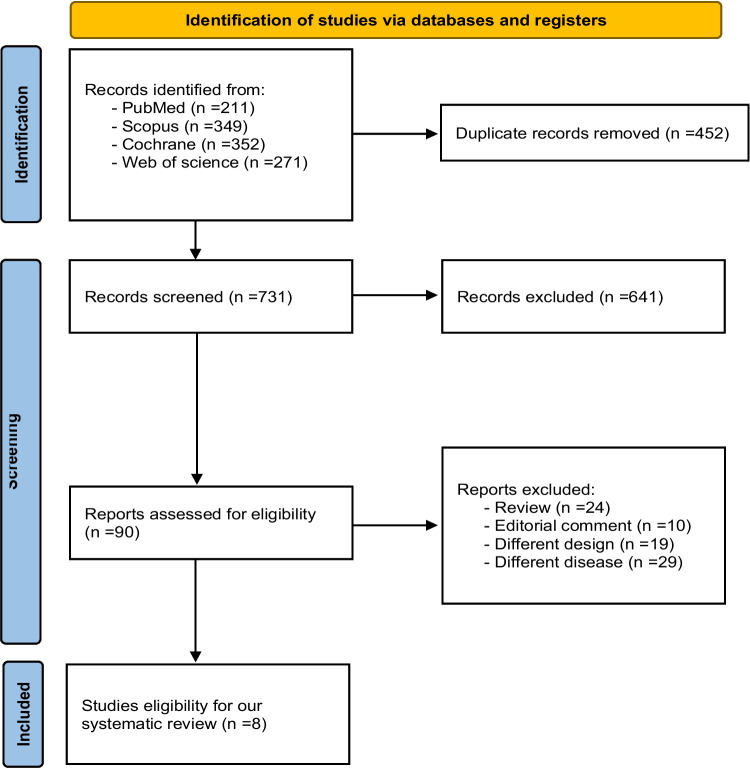
Prisma flow chart

A total of 1183 records were identified. After duplicate removal, 731 studies were reviewed based on their titles and abstracts. A total of 90 studies were deemed relevant and underwent full-text screening. Finally, eight studies were included (Sprigg et al. 2018; Yassi et al. 2024; Arumugam et al. 2023; Arumugam et al. 2015; Liu et al. 2021; Meretoja et al. 2020; Polymeris et al. 2023; Sprigg et al. 2014). The PRISMA flowchart is shown in Fig. 1.

## Study characteristics

Eight RCTs, with 2974 participants with sICH, were included in this meta-analysis. Studies were conducted across various countries, including Malaysia, China, the UK, Switzerland, Italy, Hungary, Denmark, Georgia, Ireland, Poland, Spain, Sweden, New Zealand, Taiwan, Turkey, Vietnam, Australia, Finland, and Taiwan. The intervention generally consisted of the intravenous bolus injection of 1 g TXA with a subsequent infusion of 1 to 2 g over 8 to 12 h, per study protocol. Two studies specifically targeted hypertensive ICH (Arumugam et al. 2023; Liu et al. 2021), one study focused on ICH associated with NOACs (Polymeris et al. 2023), one study focused on sICH with a spot sign (patients at higher risk of hematoma progression) (Meretoja et al. 2020), and four studies included patients without specifying specific subgroups of sICH (Sprigg et al. 2018; Yassi et al. 2024; Arumugam et al. 2015; Sprigg et al. 2014). TXA was administered within a time window of 2 h in one study (Yassi et al. 2024), 4.5 h in one study (Meretoja et al. 2020), 8 h in four studies (Sprigg et al. 2018; Arumugam et al. 2023; Arumugam et al. 2015; Liu et al. 2021), 12 h in one study (Polymeris et al. 2023), and 24 h in one study (Sprigg et al. 2014). More details are demonstrated in Table 1.

**Table 1:** Summary of included studies

Study ID	Protocol number	Country	Sample size	Intervention details	Disease type	Duration to begin intervention	Follow-up duration	Primary outcomes
Arumugam et al. 2015	NMRR-12–285-11,650	1 country (Malaysia)	30	Intravenous (1 g as a bolus, followed by 1 g/h infusion for 8 h)	Spontaneous intracerebral hemorrhage	Within 8 h of symptom onset	After 24 h	Hematoma growth
Arumugam et al. 2023	NMRR-16–995-30,649	1 country (Malaysia)	60	Intravenous 2 g (1 g of TXA as a slow bolus followed by 1 g infusion) or intravenous 3 g (1 g as a slow bolus followed by 2 g infusion)	Hypertensive intracerebral hemorrhage	Within 8 h of symptom onset	4 weeks	Hematoma growth
Liu et al. 2021	NCT02625948	1 country (China)	171	Intravenous 2 g (1 g in 100 mL 0.9% NaCl, followed by 1 g in 250 mL 0.9% NaCl infusion)	Hypertensive intracerebral hemorrhage	Within 8 h of symptom onset	12 weeks	Hematoma growth
Meretoja et al. 2020	NCT01702636	3 countries (Australia, Finland, Taiwan)	100	Intravenous 2 g	Spontaneous intracerebral hemorrhage with spot sign	Within 4.5 h of symptom onset	12 weeks	Intracerebral hemorrhage growth
Polymeris et al. 2023	NCT02866838	1 country (Switzerland)	63	Intravenous 2 g	NOAC-associated intracerebral hemorrhage	Within 12 h of symptom onset	12 weeks	Hematoma growth
Sprigg et al. 2014	ISRCTN50867461	1 country (UK)	24	Intravenous 2 g (1 g as a loading dose, followed by 1 g)	Spontaneous intracerebral hemorrhage	Within 24 h of symptom onset	12 weeks	Hematoma growth
Sprigg et al. 2018	ISRCTN93732214	12 countries (UK, Denmark, Georgia, Hungary, Ireland, Italy, Malaysia, Poland, Spain, Sweden, Switzerland, and Turkey)	2325	Intravenous 2 g (1 g as a bolus, followed by 1 g)	Spontaneous intracerebral hemorrhage	Within 8 h of symptom onset	12 weeks	Functional status at day 90
Yassi et al. 2024	NCT03385928	4 countries (Australia, Finland, New Zealand, Taiwan, and Vietnam)	202	Intravenous 2 g	Spontaneous intracerebral hemorrhage	Within 2 h of symptom onset	12 weeks	Hematoma growth

Participants were aged between 52 and 82, primarily male. The positivity of the spot sign differed across studies, with many indicating notable frequencies. The severity of strokes (as measured by NIHSS) showed similarities between the TXA and control groups, although minor variations were observed. Admission score on the Glasgow Coma Scale demonstrated moderate neurological impairment. A history of conditions such as hypertension (43 to 90%), diabetes, and previous strokes/TIAs was prevalent. Most cohorts exhibited elevated baseline systolic and diastolic blood pressures, consistent with the typical presentations of acute hemorrhagic strokes. More details are in Table 2.

**Table 2:** Baseline characteristics of included studies

Study ID	Groups (*n*)	Age, mean (SD)	Sex (male), NO. (%)	Spot sign positive, NO. (%)	NIHSS, mean (SD)	Admission GCS, mean (SD)	Prestroke mRS score, mean (SD)	Stroke/TIA, NO. (%)	CHD/MI, NO. (%)	Hypertension, NO. (%)	Diabetes, NO. (%)	Smoking, NO. (%)	Admission SBP, mean (SD)	Admission DBP, mean (SD)
Arumugam et al. 2015	Tranexamic acid and control groups (30)	53	18 (60%)	NA	NA	14 (0.56)	NA	NA	NA	13 (43%)	8 (27%)	NA	185 (8)	90 (8)
Arumugam et al. 2023	Tranexamic acid 2 g (20)	52 (12)	11 (55%)	NA	NA	NA	NA	NA	NA	15 (75%)	1 (5%)	NA	197 (21)	NA
	Tranexamic acid 3 g (20)	52.4 (10)	13 (65%)	NA	NA	NA	NA	NA	NA	12 (60%)	1 (5%)	NA	195 (30)	NA
	Control (20)	53 (10)	12 (60%)	NA	NA	NA	NA	NA	NA	16 (80%)	4 (20%)	NA	188 (52)	NA
Liu et al. 2021	Tranexamic acid (89)	57 (12)	63 (71%)	50 (56.2%)	11 (6)	13 (3)	NA	5 (6%)	1 (1%)	64 (72%)	12 (14%)	21 (24%)	176 (28)	100 (17)
	Control (82)	55 (11)	61 (74%)	44 (53.7%)	10 (7)	13 (3)	NA	3 (4%)	2 (2%)	50 (61%)	6 (7%)	24 (29%)	171 (28)	103 (20)
Meretoja et al. 2020	Tranexamic acid (50)	69 (18)	35 (70%)	50 (100%)	13 (8)	13 (3)	0.3 (0.76)	6 (12%)	NA	36 (72%)	10 (20%)	7 (14%)	168 (25)	91 (21)
	Control (50)	69 (16)	27 (54%)	50 (100%)	12 (7)	14 (1.5)	0	5 (10%)	NA	33 (66%)	9 (18%)	6 (12%)	173 (25)	90 (17)
Polymeris et al. 2023	Tranexamic acid (32)	81 (9)	18 (56%)	3 (10%)	12 (8.5)	13 (2)	1 (2)	5 (16%)	NA	26 (81%)	7 (22%)	NA	164 (28)	93 (23)
	Control (31)	82 (6)	20 (65%)	2 (8%)	12 (12)	14 (2)	1 (1.5)	9 (29%)	NA	28 (90%)	6 (19%)	NA	172 (25)	100 (22)
Sprigg et al. 2014	Tranexamic acid (16)	68 (13)	10 (63%)	NA	15 (9)	13 (3)	0.5 (1.0)	3 (19%)	NA	10 (63%)	2 (13%)	3 (19%)	167 (20)	NA
	Control (8)	69 (13)	4 (50%)	NA	16 (9)	13 (3)	0.1 (0.4)	2 (25%)	NA	5 (63%)	0	0	166 (28)	NA
Sprigg et al. 2018	Tranexamic acid (1161)	69 (14)	642 (55%)	24 (20%)	13 (7.5)	13 (2)	0.3 (0.7)	173 (15%)	110 (10%)	NA	NA	NA	172 (28)	93 (18)
	Control (1164)	69 (14)	659 (57%)	32 (25%)	13 (7.5)	14 (2)	0.3 (0.7)	156 (14%)	92 (8%)	NA	NA	NA	174 (27)	94 (18)
Yassi et al. 2024	Tranexamic acid (103)	65 (17)	63 (61%)	NA	13 (6)	15 (7)	0	10 (10%)	NA	68 (66%)	15 (15%)	18 (17%)	168 (25)	90 (15)
	Control (98)	67 (15)	56 (57%)	NA	13 (8)	14 (2)	0	8 (8%)	NA	60 (61%)	21 (21%)	9 (9%)	163 (21)	91 (20)

## Meta-analysis

### Hematoma expansion

**Fig. 2:**
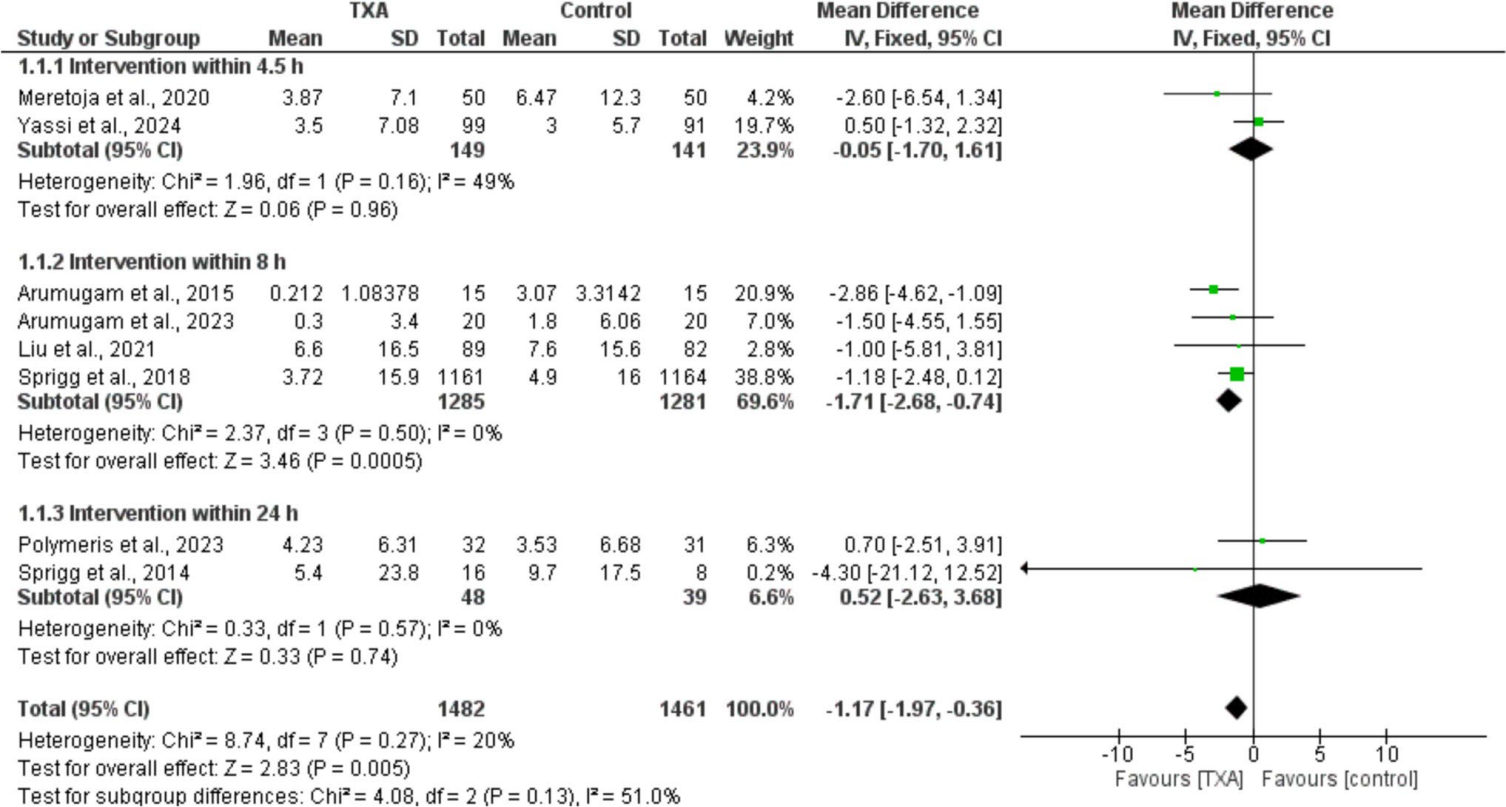
Forest plots comparing the absolute change in hematoma volume (mL), measured 24 h after the event, across different TXA administration time windows

As shown in Fig. 2, The TXA group exhibited a significantly smaller volume expansion of the hematoma after 24 h of the event than the placebo group [MD =  − 1.17, 95% CI =  − 1.97 to − 0.36, *p* = 0.005]. This significant reduction is primarily attributed to the subgroup of studies administration TXA within 8 h [MD =  − 1.71, 95% CI =  − 2.68 to − 0.74, *p* = 0.0005]. In contrast, no significant differences were observed in the 4.5-h or within 24-h subgroups: [MD =  − 0.05, 95% CI =  − 1.7 to 1.61] and [MD = 0.52, 95% CI =  − 2.63 to 3.68], respectively. Another subgroup analysis based on study populations revealed a significant effect of TXA on hematoma volume in studies involving sICH without specific subgroup classification [MD =  − 1.22, 95% CI =  − 2.12 to − 0.31, *p* = 0.008]. However, no significant effect was observed in other subgroups, including sICH with spot sign, hypertensive ICH, and NOAC-associated ICH (Supplementary Fig. 1). No significant heterogeneity was detected across all analyses.

**Fig. 3:**
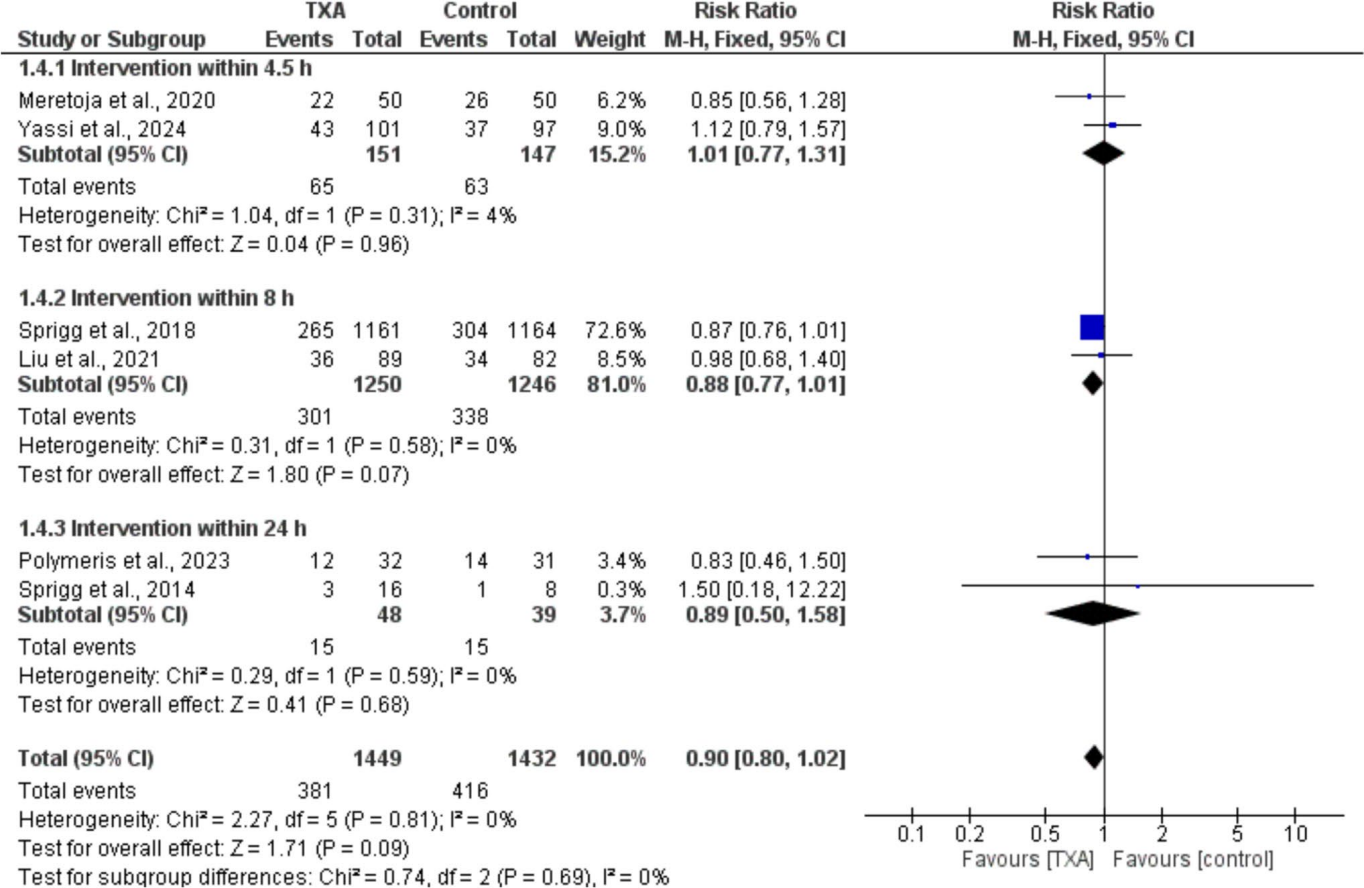
Forest plots comparing the odds of hematoma expansion in TXA and placebo groups across different TXA administration time windows

Regarding the odds of hematoma expansion, no significant difference was observed between TXA and placebo [RR = 0.90, 95% CI = 0.8 to 1.02, *p* = 0.09]. The subgroup analysis based on the time window of TXA administration (4.5 h, 8 h, and 24 h) did not reveal any statistically significant differences between the groups (Fig. 3). Furthermore, no significant differences in the odds of hematoma expansion were observed between the TXA and placebo groups across different study population subgroups (Supplementary Fig. 2). No significant heterogeneity was detected across all analyses.

### Functional outcomes

**Fig. 4:**
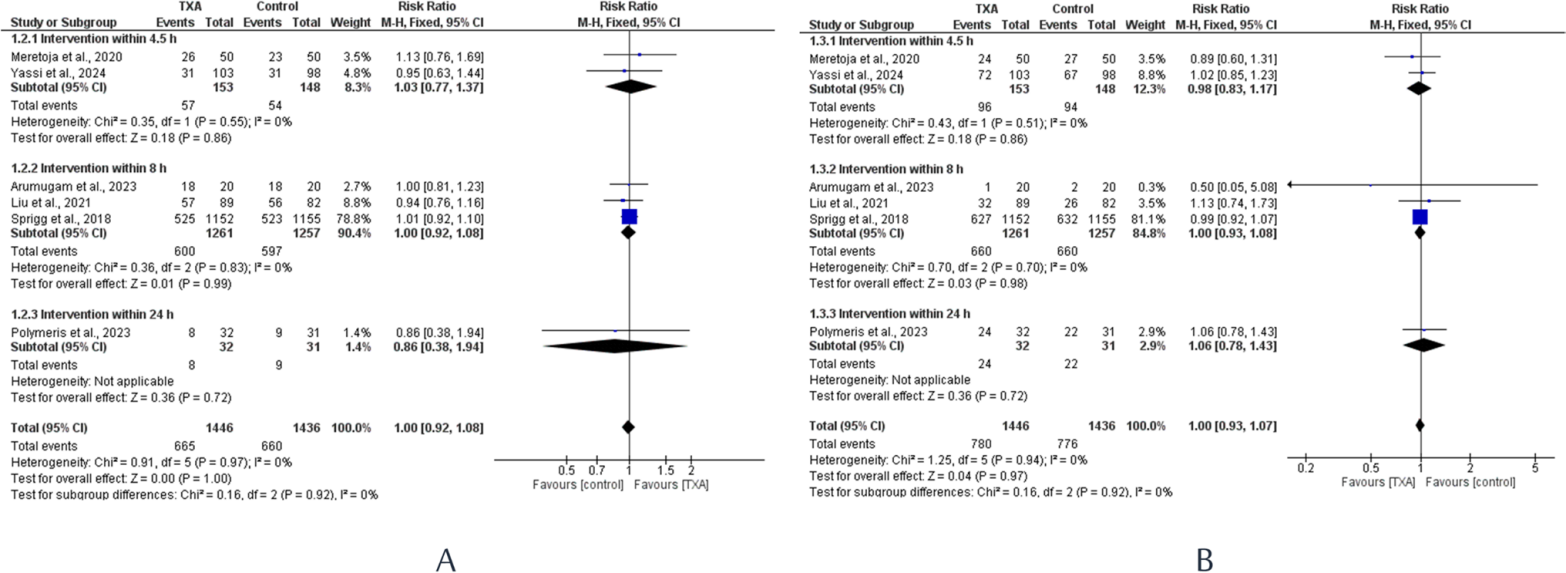
Forest plots comparing the modified Rankin Scale (mRS) between TXA and placebo groups across different time windows. **A** presents the odds of patients achieving mRS scores 0 to 3, while **B** illustrates the odds of patients achieving mRS scores 4 to 6

No significant differences were observed regarding the functional outcomes between TXA and placebo groups. The odds of patients achieving mRS scores 0 to 3 and 4 to 6 were comparable between both groups: [RR = 1.00, 95% CI = 0.92 to 1.08] and [RR = 1.00, 95% CI = 0.93 to 1.07]. After subgrouping based on time windows, TXA continued to show no significant effect (Fig. 4A, B). Similarly, subgrouping based on study populations yielded no significant differences across all subgroups (Supplementary Figs. 3 and 4).

**Fig. 5:**
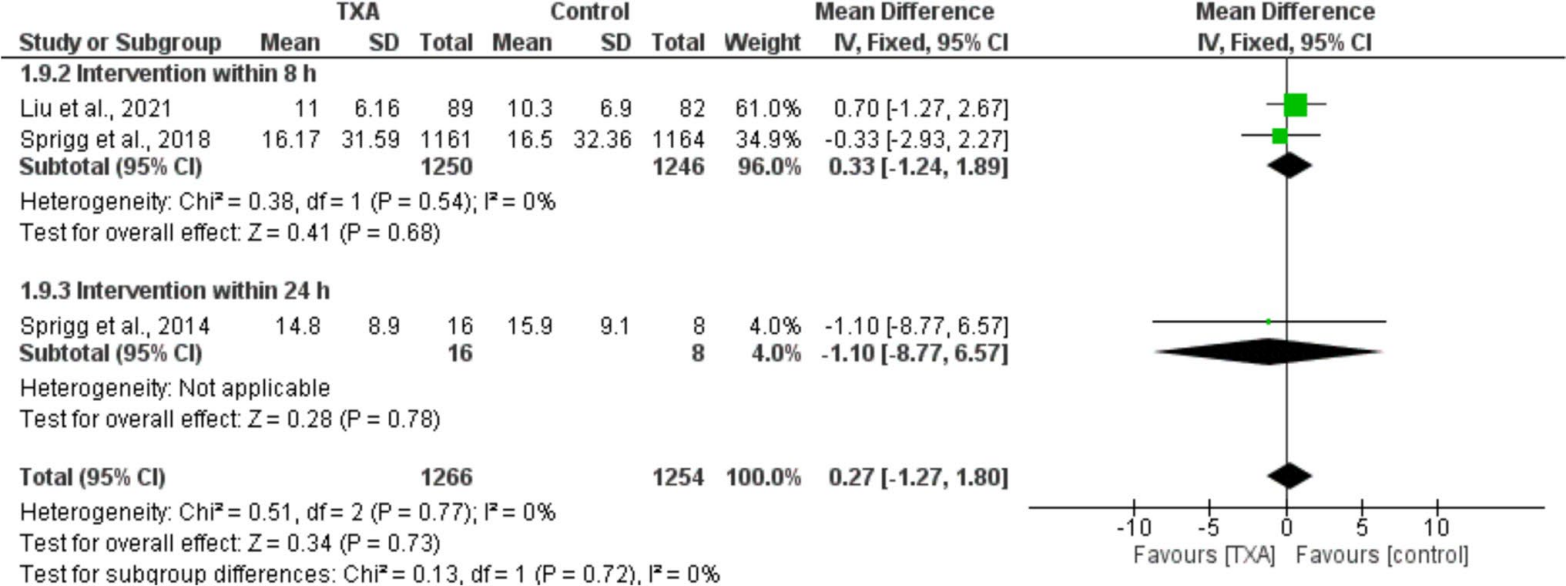
Forest plots comparing the NIHSS scores in TXA and placebo groups across different TXA administration time windows

Regarding NIHSS score, no significant differences were noted between TXA and placebo groups [MD = 0.27, 95% CI =  − 1.27 to 1.80]. Similarly, subgrouping based on time windows *(*Fig. 5*)* and study populations (Supplementary Fig. 5) yielded no significant differences across all subgroups. No significant heterogeneity was detected across all analyses.

### Mortality within 90 days

**Fig. 6:**
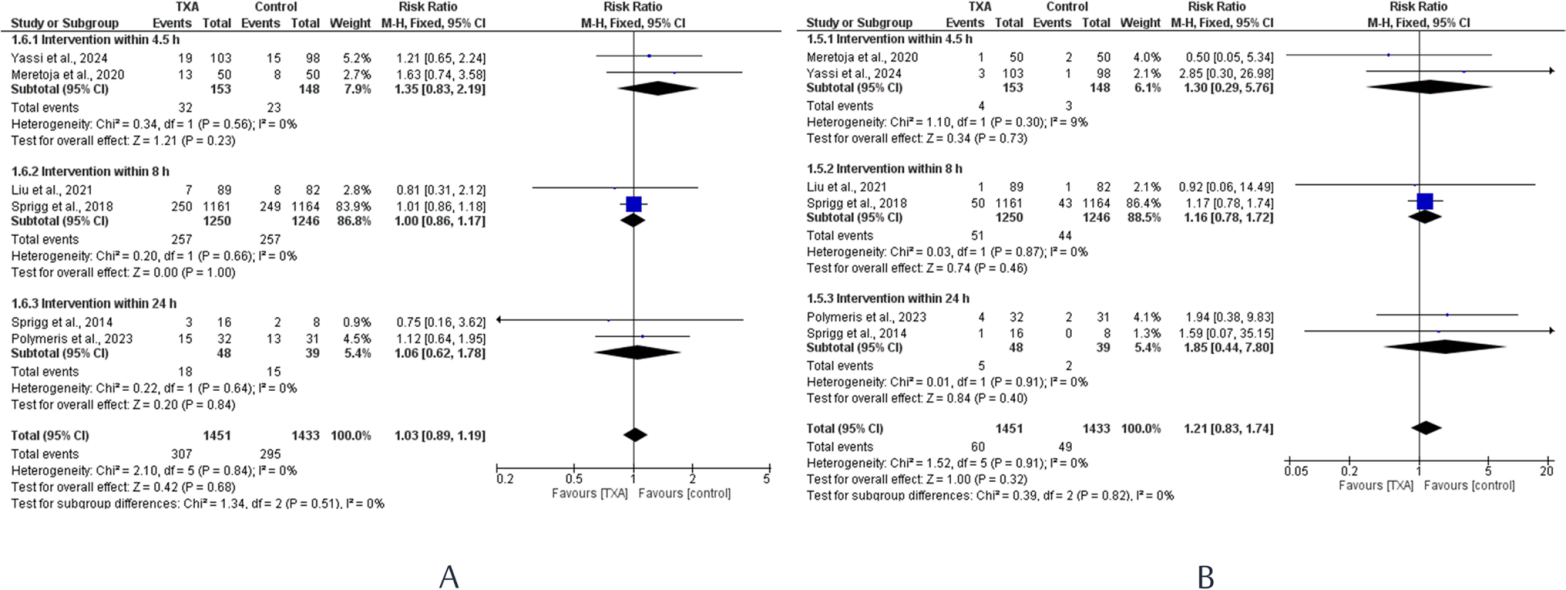
Forest plots comparing safety outcomes between the TXA and placebo groups across different TXA administration time windows. **A** shows the odds of mortality within 90 days, while **B** illustrates the odds of major thromboembolic events

Mortality rates within 90 days of the event were comparable between TXA and placebo groups [RR = 1.03, 95% CI = 0.89 to 1.19]. Subgrouping based on time windows *(*Fig. 6A*)* and study populations (Supplementary Fig. 6) yielded no significant difference across all subgroups. No significant heterogeneity was detected across all analyses. No significant heterogeneity was detected across all analyses.

### Major thromboembolic events

The risk of major thromboembolic events was similar between the TXA and placebo groups [RR = 1.21, 95% CI = 0.83 to 1.74]. Subgroup analyses based on time windows *(*Fig. 6B*)* and study populations (Supplementary Fig. 7) revealed no significant differences across subgroups. Additionally, no significant heterogeneity was observed in any of the analyses.

### Health-related quality of life

**Fig. 7:**
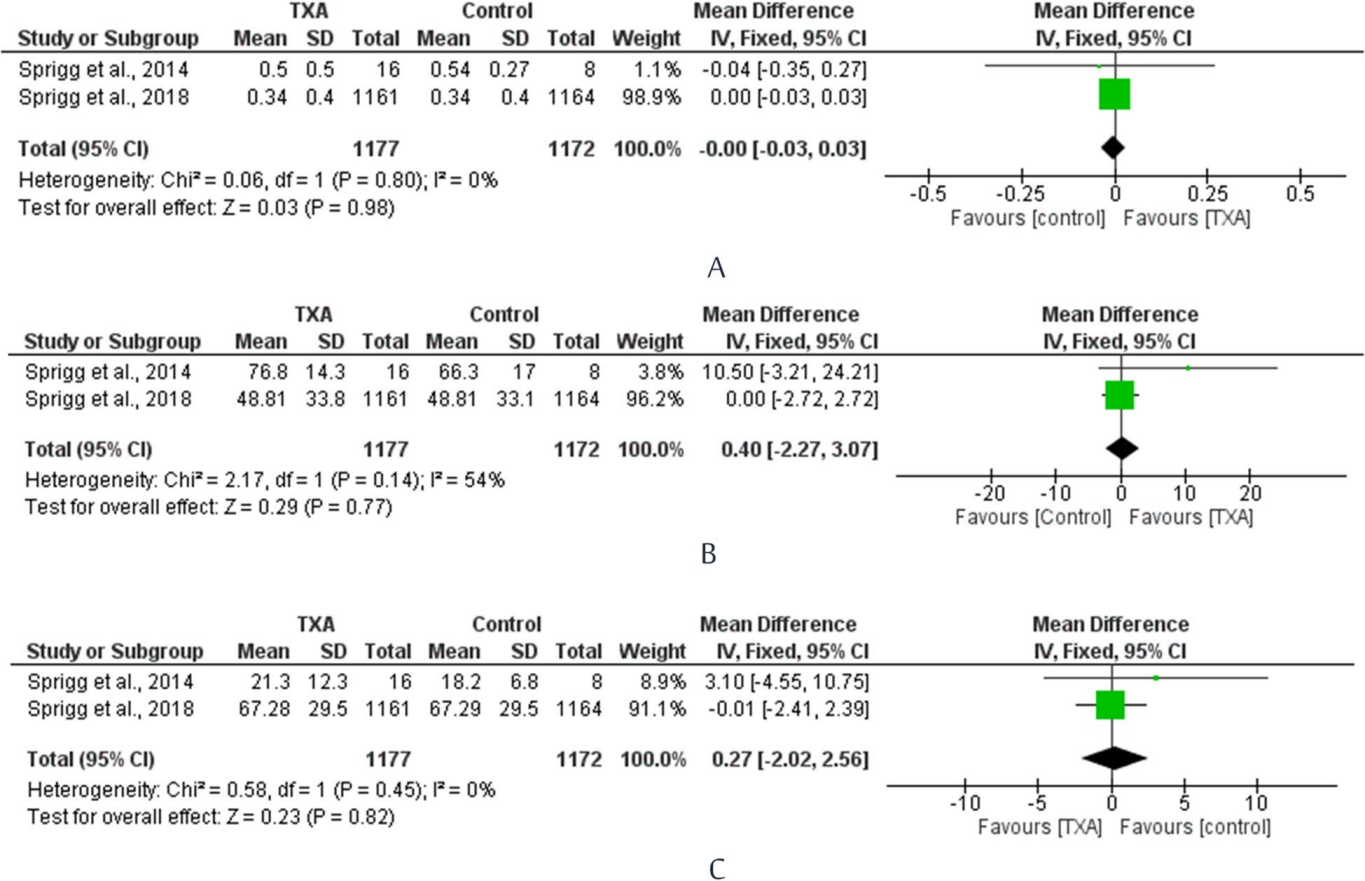
Forest plots comparing health-related quality of life in TXA and placebo groups in terms of EQ-5D HUS (A), EQ-VAS (B), and ZDS (C)

As shown in Fig. 7A, B, and C, no significant differences were noted in terms of EQ-5D HUS, EQ-VAS, and ZDS: [MD =  − 0.00, 95% CI =  − 0.03 to 0.03], [MD = 0.4, 95% CI =  − 2.27 to 3.07], and [MD = 0.27, 95% CI =  − 2.02 to 2.56], respectively.

### Quality assessment

**Fig. 8:**
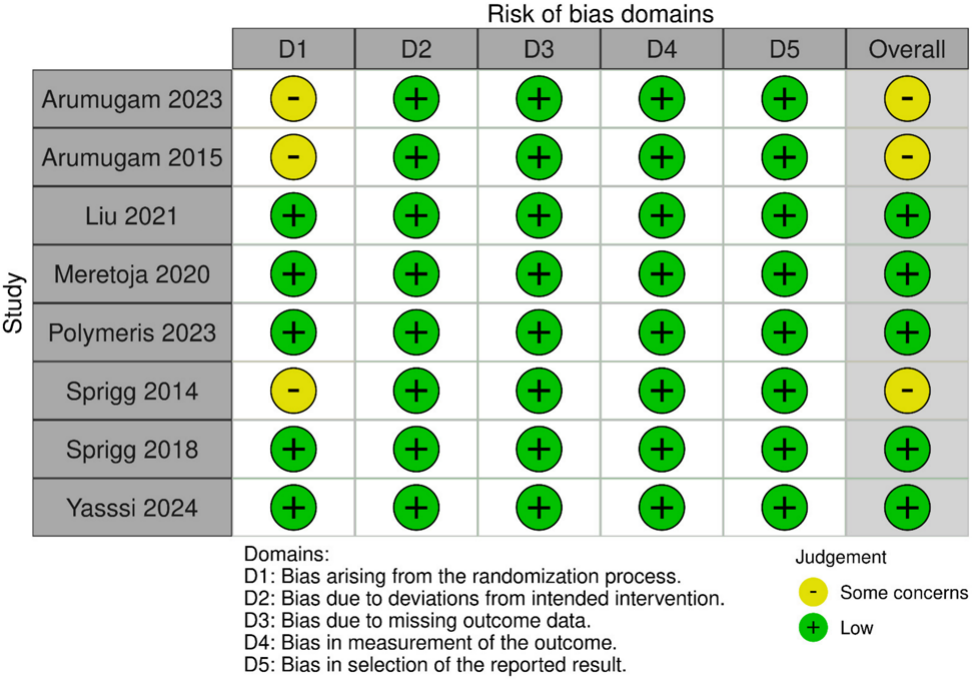
Cochrane risk-of-bias tool (ROB 2)

Most studies demonstrated a low risk of bias across multiple domains. The studies by Arumugam et al. 2015, Arumugam et al. (2023), and Sprigg et al. 2014 presented some concerns, particularly in the domain of the randomization process. Results of the risk of bias are demonstrated in Fig. 8.

## Discussion

The findings of this meta-analysis demonstrate that TXA is associated with a small but significant reduction in hematoma volume in patients with sICH, mainly when administered within 8 h of symptom onset, suggesting a time-sensitive therapeutic window for TXA efficacy. However, the subgroup focusing on earlier time windows of TXA administration (within 4.5 h) did not show a significant effect. Another subgroup analysis based on study populations revealed a significant effect of TXA on hematoma volume in studies involving sICH without a specific subgroup classification. However, no significant effect was observed in other subgroups, including sICH with spot sign, hypertensive ICH, and NOAC-associated ICH. Furthermore, no significant differences were noted in terms of the odds of hematoma expansion across the different time windows and subgroups of study populations. The reduction in hematoma volume did not translate into significant improvements in functional outcomes, 90-day mortality rates, or health-related quality of life.

The correlation between hematoma expansion and clinical outcomes is established; however, the cutoff values are debatable. Dowlatshahi and colleagues reported that while all hematoma expansion definitions predicted poor outcomes, absolute growth was more predictive than relative growth (Dowlatshahi et al. 2011). In our analysis, although TXA was associated with a statistically significant reduction in hematoma volume (mean difference: − 1.17 mL), this modest absolute reduction may fall below the threshold required to produce meaningful clinical or functional benefits. This aligns with recent findings showing that the magnitude of hematoma resolution is critical to functional outcomes (Malinova et al. 2025). Specifically, patients achieving a ≥ 50% reduction in hematoma volume within 24 h had four times higher odds of favorable outcomes, while those with residual hematoma volumes < 30 mL at 24 h had nearly twice the likelihood of recovery. These data suggest that although TXA may effectively limit hematoma expansion, the degree of reduction observed in our pooled analysis is likely insufficient to significantly alter clinical trajectories.

Previous meta-analysis studies have focused on the same topic. Guo and colleagues (Guo et al. 2021) concluded that TXA may reduce the risk of hematoma expansion in acute sICH, particularly in high-risk patients with CT markers of hematoma expansion and those treated within 4.5 h of symptom onset. This particular improvement in these subgroups does not go in line with our findings. While their findings suggested a potential benefit in these specific populations, the magnitude of the observed effect was modest and did not clearly translate into improved functional outcomes. In contrast, our updated meta-analysis did not replicate this subgroup-specific benefit. A likely reason for this discrepancy is the inclusion of more recent data, particularly the large-scale study by Yassi 2024, which contributed substantially to the overall weight and attenuated the previously observed effect in early-presenting or high-risk patients. Nevertheless, consistent with Guo et al., our pooled results showed no significant difference between TXA and placebo in terms of 3-month functional outcomes, mortality, or thromboembolic events.

Delaying administration of TXA in acute severe hemorrhage was reported to be linked with a decrease in survival benefits. A previous meta-analysis stated that every 15-min delay in TXA administration is associated with a decrease of 10% in survival benefits until 3 h, after which there was no benefit (Gayet-Ageron et al. 2018). Meretoja et al. 2020 studied early TXA administration within 4.5 h. Although TXA is safe, no evidence has been identified that it can prevent hematoma expansion. The recent trial (STOP-MSU) tested the efficacy of TXA administration within the shortest time window (2 h). Similarly, they did not report significant effects on hematoma expansion, functional outcomes, or safety outcomes (Yassi et al. 2024). However, these trials are limited by small sample sizes. Further trials with larger sample sizes are needed to investigate and compare the role of earlier time windows.

Spot signs independently predict ICH growth and poor outcomes (Demchuk et al. 2012). The only trial in this meta-analysis (STOP-AUST) that included only sICH with a positive spot sign did not show any evidence of the efficacy of TXA (Meretoja et al. 2020). A post hoc analysis of the TICH-2 trial did not find significant differences in hematoma expansion outcomes between spot-sign positive and spot-sign negative groups (Ovesen et al. 2021). However, the most recent individual patient meta-analysis by Yassi and colleagues (Yassi et al. 2024) detected a significant reduction in the absolute increase of hematoma volume in TXA when administered within 4.5 h. Additionally, they reported a substantial difference in the odds of hematoma expansion. Similarly, this was not translated into improved functional outcomes or safety differences. The difference in significance in this subgroup (sICH with spot sign) between their analysis and ours likely stems from their inclusion of a larger dataset of individual patient data, allowing for greater statistical power to detect these effects.

This systematic review and meta-analysis provide an updated assessment of the role of TXA in sICH, pooling the evidence from all available RCTs. The included studies were subgrouped based on the time windows of TXA administration and study-specific populations. The largest trial, TICH-2, which included 2325 patients and accounted for approximately 80% of the total sample in this meta-analysis, heavily influenced the overall findings (Sprigg et al. 2018). Although the included studies are highly consistent and our results are statistically homogenous, we should acknowledge some limitations. The small number of studies in some subgroups does not allow the actual effects to be judged. Despite including only RCTs, there was slight variability in study protocols, including differences in population, TXA dose, follow-up, baseline hematoma volume, baseline blood pressure, and timing of TXA administration. Different subgroup analyses addressed this. Another limitation is that we could not subgroup studies based on baseline hematoma volume or blood pressure. A previous meta-analysis stated that TXA was more efficacious in patients with moderate to severe hypertension. However, this meta-analysis included both traumatic and non-traumatic ICH (Gao et al. 2021). The dose examined in this meta-analysis was primarily 2 g of TXA, administered as 1 g for loading and one gram for maintenance. However, higher doses, such as the 3-g regimen explored in the TANICH II trial, were not included, as this approach has only been investigated in a single trial to date. The TANICH II trial suggested that a 3-g dose might more effectively reduce hematoma volume rather than merely prevent expansion. Although these findings did not reach statistical significance, they are promising and warrant further investigation in larger, well-designed trials to evaluate the potential benefits of higher TXA doses.

## Conclusion

TXA has been shown to reduce hematoma expansion in sICH, particularly when administered within 8 h of symptom onset, without increasing the risk of thromboembolic complications. Despite this radiological benefit, current evidence indicates that TXA does not significantly improve functional outcomes, 90-day mortality, or quality of life. While TXA appears to be a safe intervention, existing data do not support its routine use in the management of sICH. Further research is needed to better define the clinical benefit of TXA, identify subgroups of patients most likely to respond, and determine the optimal timing and dosing strategy for treatment.

## Supplementary Information

Below is the link to the electronic supplementary material.

Supplementary file1 (DOCX 188 KB)

## Data Availability

All source data for this work (or generated in this study) are available upon reasonable request.
