# Supplementary material for: Tranexamic acid for spontaneous intracerebral hemorrhage: hematoma control without clinical benefits? A meta-analysis of RCTs
Source: Naunyn Schmiedebergs Arch Pharmacol. 2025 Jul 28;399(1):1109–20. doi: 10.1007/s00210-025-04473-5 (PMC12894203; doi:10.1007/s00210-025-04473-5)
Supplement: Supplementary file 1 — Supplementary file1 (DOCX 188 KB) [file 210_2025_4473_MOESM1_ESM.docx]

**Title: The Impact of Tranexamic Acid on Spontaneous Intracerebral Hemorrhage: A Systematic Review and Meta-Analysis of RCTs.**

**Journal: *CNS Drugs***

**Authors:**

Mahmoud M. Elhady^1*^, Eslam Mohammed Rabea^2^, Samah Bahy Mohammed Ebaed^3^, Manar Adel^4^, Moustafa Z. Elattar^5^, Mahmoud Eleisawy^6^, Ahmed A. Lashin^1^, Ahmed A. Elfeky^7^, Mohamed Hesham Gamal^8^, Mohamed Sayed Zaazouee^9^.

**Affiliations:**

1- Faculty of Medicine, Benha University, Qalubiya, Egypt.

2- Faculty of Medicine, Alexandria University, Alexandria, Egypt.

3- Department of Clinical Pharmacology, Faculty of Medicine, Benha University, Egypt.

4- Faculty of Clinical Pharmacy, Tanta University, Gharbia, Egypt.

5- Anatomy & Embryology Department, Faculty of Medicine, Benha University, Qalubiya, Egypt.

6- Ophthalmology Department, Benha University Hospitals, Qalubiya, Egypt.

7- Faculty of Medicine, Beni Suef University, Beni Suef, Egypt.

8- Faculty of Pharmacy, Tanta University, Gharbia, Egypt.

9- Faculty of Medicine, Al-Azhar University, Assiut, Egypt.

***Corresponding author**

**Name:** Mahmoud M. Elhady

**Email:** mahmoud160375@fmed.bu.edu.eg

**Supplementary Table 1.** Search strategies across the four databases.

| Database | Strategy | Results |
| --- | --- | --- |
| Scopus | TITLE-ABS-KEY ( ( intracerebral OR subarachnoid OR intraparenchymal OR parenchymal OR intraventricular OR intra-axial OR intracranial OR brain OR cerebr* OR stroke* ) AND ( hemorrhag* OR bleed* OR hematoma ) AND ( "Tranexamic acid" OR amca OR amcha OR t-amcha OR "trans-4-(Aminomethyl)cyclohexanecarboxylic Acid" OR cyklokapron OR ugurol OR transamin OR kabi 2161 OR amchafibrin OR anvitoff OR spotof OR exacyl OR lysteda OR evana OR antifibrinolytic ) AND ( random* ) ) | 349 |
| Pubmed | (Intracerebral[Title/Abstract] OR Subarachnoid[Title/Abstract] OR Intraparenchymal[Title/Abstract] OR parenchymal[Title/Abstract] OR Intraventricular[Title/Abstract] OR intra-axial[Title/Abstract] OR Intracranial[Title/Abstract] OR brain[Title/Abstract] OR Cerebr*[Title/Abstract] OR Stroke*[Title/Abstract]) AND (hemorrhag*[Title/Abstract] OR bleed*[Title/Abstract] OR hematoma[Title/Abstract]) AND ("Tranexamic acid"[Title/Abstract] OR AMCA[Title/Abstract] OR AMCHA[Title/Abstract] OR t-AMCHA[Title/Abstract] OR "trans-4-(Aminomethyl)cyclohexanecarboxylic Acid"[Title/Abstract] OR Cyklokapron[Title/Abstract] OR Ugurol[Title/Abstract] OR Transamin[Title/Abstract] OR KABI 2161[Title/Abstract] OR Amchafibrin[Title/Abstract] OR Anvitoff[Title/Abstract] OR Spotof[Title/Abstract] OR Exacyl[Title/Abstract] OR Lysteda[Title/Abstract] OR Evana[Title/Abstract] OR antifibrinolytic[Title/Abstract]) AND (random*[Title/Abstract]) | 211 |
| Cochrane | ((Intracerebral OR Subarachnoid OR Intraparenchymal OR parenchymal OR Intraventricular OR intra-axial OR Intracranial OR brain OR Cerebr* OR Stroke*) AND (hemorrhag* OR bleed* OR hematoma) AND (“Tranexamic acid” OR AMCA OR AMCHA OR t-AMCHA OR “trans-4-(Aminomethyl)cyclohexanecarboxylic Acid” OR Cyklokapron OR Ugurol OR Transamin OR KABI 2161 OR Amchafibrin OR Anvitoff OR Spotof OR Exacyl OR Lysteda OR Evana OR antifibrinolytic) AND (random*)):ti,ab,kw | 352 |
| WOS | TS=((Intracerebral OR Subarachnoid OR Intraparenchymal OR parenchymal OR Intraventricular OR intra-axial OR Intracranial OR brain OR Cerebr* OR Stroke*) AND (hemorrhag* OR bleed* OR hematoma) AND (“Tranexamic acid” OR AMCA OR AMCHA OR t-AMCHA OR “trans-4-(Aminomethyl)cyclohexanecarboxylic Acid” OR Cyklokapron OR Ugurol OR Transamin OR KABI 2161 OR Amchafibrin OR Anvitoff OR Spotof OR Exacyl OR Lysteda OR Evana OR antifibrinolytic) AND (random*)) | 271 |


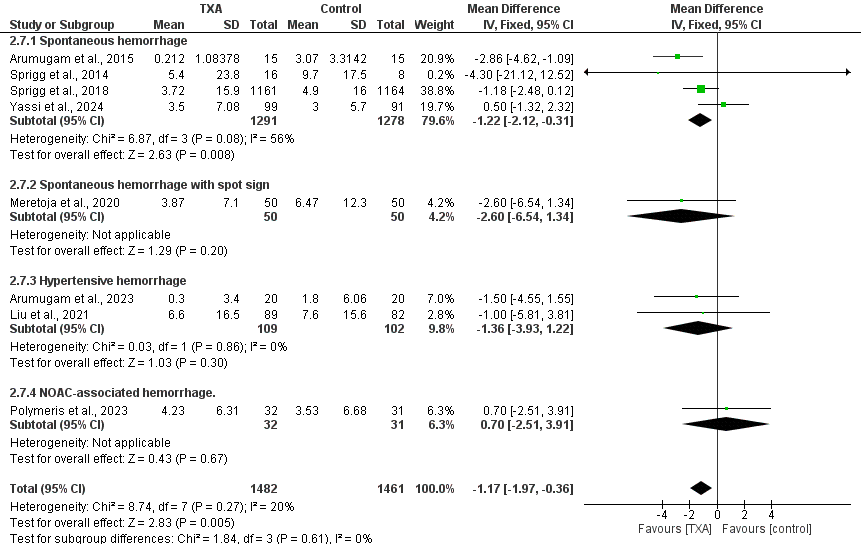


**Supplementary Figure 1.** Forest plots comparing the absolute change in hematoma volume (mL), measured 24 hours after the event, across different subgroups of studies’ populations.


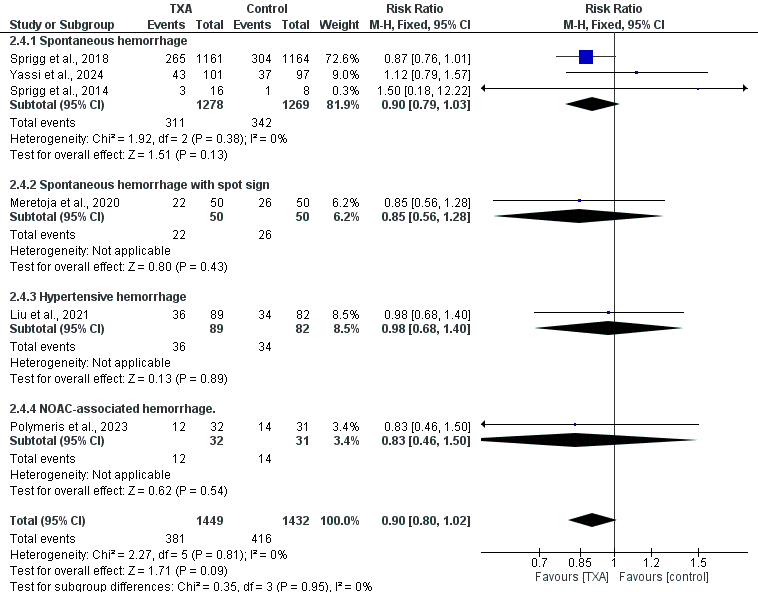


**Supplementary Figure 2.** Forest plots comparing the odds of hematoma expansion across different subgroups of studies’ populations.


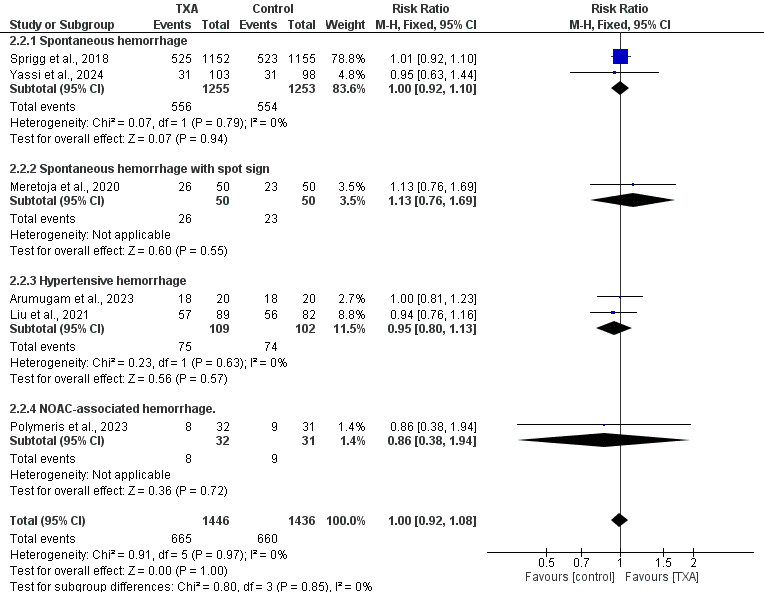


**Supplementary Figure 3**. Forest plots comparing the odds of patients achieving mRS score 1 to 3 across different subgroups of studies’ populations.


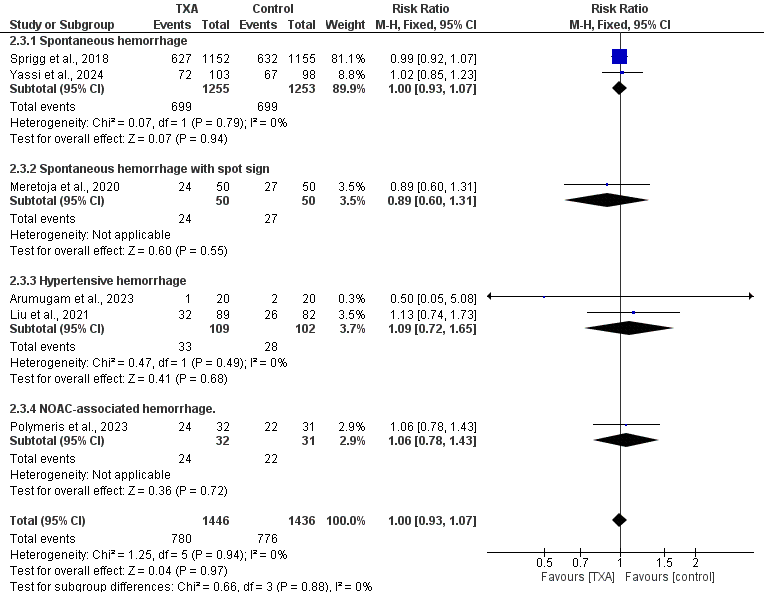


**Supplementary Figure 4**. Forest plots comparing the odds of patients achieving mRS score 4 to 6 across different subgroups of studies’ populations.


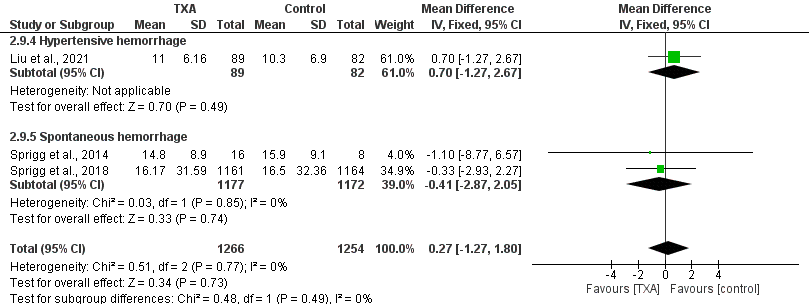


**Supplementary Figure 5**. Forest plots comparing NIHSS between TXA and placebo groups across different subgroups of studies’ populations.


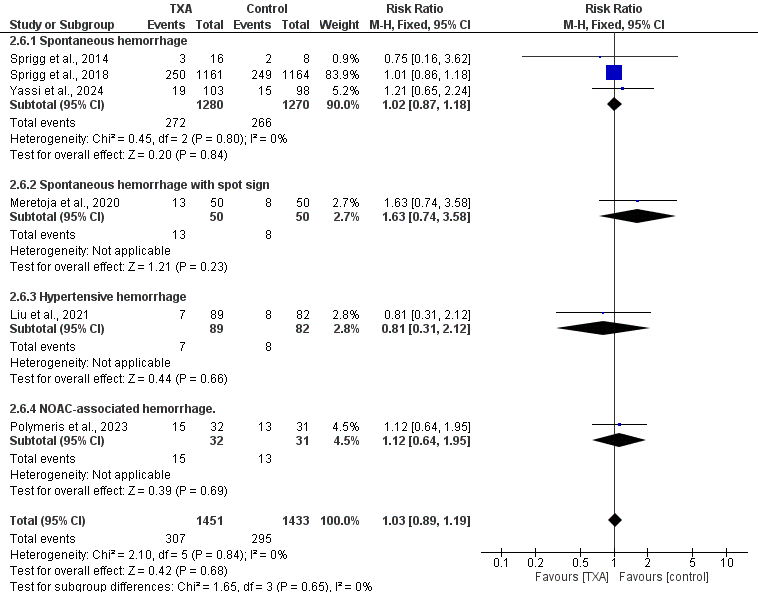


**Supplementary Figure 6**. Forest plots comparing mortality within 90 days between TXA and placebo groups across different subgroups of studies’ populations.


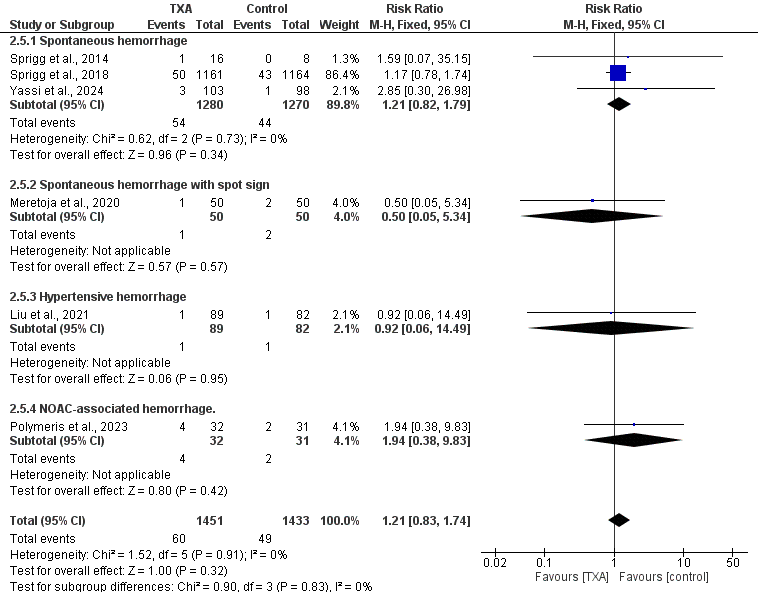


**Supplementary Figure 7**. Forest plots comparing the odds of major thromboembolic events between TXA and placebo groups across different subgroups of studies’ populations.
